# Effect of Age on Complexity and Causality of the Cardiovascular Control: Comparison between Model-Based and Model-Free Approaches

**DOI:** 10.1371/journal.pone.0089463

**Published:** 2014-02-24

**Authors:** Alberto Porta, Luca Faes, Vlasta Bari, Andrea Marchi, Tito Bassani, Giandomenico Nollo, Natália Maria Perseguini, Juliana Milan, Vinícius Minatel, Audrey Borghi-Silva, Anielle C. M. Takahashi, Aparecida M. Catai

**Affiliations:** 1 Department of Biomedical Sciences for Health, University of Milan, Milan, Italy; 2 Galeazzi Orthopedic Institute, Milan, Italy; 3 Department of Physics and BIOtech, University of Trento, Trento, Italy; 4 Gruppo Ospedaliero San Donato Foundation, Milan, Italy; 5 Department of Electronics Information and Bioengineering, Politecnico di Milano, Milan, Italy; 6 Department of Anesthesia and Intensive Care, Humanitas Clinical and Research Center, Rozzano, Italy; 7 Humanitas Clinical and Research Center, Rozzano, Italy; 8 BIOtech, Department of Industrial Engineering, University of Trento, Trento, Italy; 9 IRCS PAT-FBK, Trento, Italy; 10 Department of Physiotherapy, Federal University of São Carlos, São Carlos, São Paulo State, Brazil; University of Adelaide, Australia

## Abstract

The proposed approach evaluates complexity of the cardiovascular control and causality among cardiovascular regulatory mechanisms from spontaneous variability of heart period (HP), systolic arterial pressure (SAP) and respiration (RESP). It relies on construction of a multivariate embedding space, optimization of the embedding dimension and a procedure allowing the selection of the components most suitable to form the multivariate embedding space. Moreover, it allows the comparison between linear model-based (MB) and nonlinear model-free (MF) techniques and between MF approaches exploiting local predictability (LP) and conditional entropy (CE). The framework was applied to study age-related modifications of complexity and causality in healthy humans in supine resting (REST) and during standing (STAND). We found that: 1) MF approaches are more efficient than the MB method when nonlinear components are present, while the reverse situation holds in presence of high dimensional embedding spaces; 2) the CE method is the least powerful in detecting age-related trends; 3) the association of HP complexity on age suggests an impairment of cardiac regulation and response to STAND; 4) the relation of SAP complexity on age indicates a gradual increase of sympathetic activity and a reduced responsiveness of vasomotor control to STAND; 5) the association from SAP to HP on age during STAND reveals a progressive inefficiency of baroreflex; 6) the reduced connection from HP to SAP with age might be linked to the progressive exploitation of Frank-Starling mechanism at REST and to the progressive increase of peripheral resistances during STAND; 7) at REST the diminished association from RESP to HP with age suggests a vagal withdrawal and a gradual uncoupling between respiratory activity and heart; 8) the weakened connection from RESP to SAP with age might be related to the progressive increase of left ventricular thickness and vascular stiffness and to the gradual decrease of respiratory sinus arrhythmia.

## Introduction

The spontaneous fluctuations of heart period (HP) about its mean value observable in five minutes’ recordings are the apparent manifestation of the short-term cardiovascular control [1], [2]. Short-term cardiovascular regulation is carried out by a set of interacting neural and non neural components simultaneously operating over a range of frequencies from 0.04 to 0.5 Hz in humans [3]. Since these regulatory mechanisms work according to similar but not coincident temporal scales and they are coordinated by the autonomic nervous system but maintain a certain degree of autonomy to accomplish specific local tasks (e.g. the maintenance of the peripheral vasomotion at the district level in presence of vasoconstriction), the dynamics of HP changes cannot be fully described by a finite number of strictly periodic, fully predictable, oscillations. Complexity analysis quantifies the departure of a given signal from a fully predictable course [4]–[11]: the smaller the predictability, the higher the complexity. The improvement of predictability of an assigned effect signal when a presumed cause is introduced in the multivariate data set has been suggested to be a measure of the strength of the causal relation from the cause to the effect [12]: the larger the predictability improvement, the strong the intensity of the cause-effect link.

It is well known that aging influences the complexity of the cardiovascular control, as assessed from the analysis of HP variability, by reducing the number of temporal scales involved into the regulatory process, especially in the high frequency band (i.e. above 0.15 Hz) [4]–[9]. This information is clinically relevant because it was suggested that complexity analysis of HP variability can provide noninvasive indexes for monitoring the aging process and the susceptibility of individuals to injury and illness [10]. Nonetheless, two main issues deserve elucidation. The first issue is related to the traditional approach to assess the influence of age on the cardiovascular control: it is almost exclusively based on the analysis of HP variability. However, recently it has been pointed out that complexity analysis of systolic arterial pressure (SAP) variability can provide additional information [11]. We hypothesize that tracking the course of complexity of SAP variability and respiration (RESP) with age can provide information closely related to senescence of vascular and respiratory systems, thus complementing the traditional view exploring solely the senescence of cardiac control according to the analysis of HP variability. The second issue is linked to the possibility provided by causality tools [12]–[16] in interpreting changes of complexity of a designated variable in terms of modifications of the strength of the relation between the variable and its determinants [17]. For example, it is well-known that SAP variability and RESP contribute to HP oscillations respectively through the cardiac baroreflex [18]–[20] and the coupling between respiratory activity and vagal outflow [18], [21]–[23]. In a more complete universe of knowledge including SAP and RESP variability in addition to the HP one, causality analysis might explain the variation of the complexity of HP variability observed, for example, during STAND [24] as the result of the variation of the strength of the causal relation from SAP to HP and/or from RESP to HP. We hypothesize that complementing traditional univariate complexity analysis with the assessment of the strength of the causal relations via causality analysis might provide a more insightful description of the evolution of the cardiovascular control with age by linking the observed change of complexity to physiological mechanisms and their impairment.

According to these hypotheses the aim of this study is to assess the influence of aging on the complexity of HP, SAP and RESP variabilities and on the strength of the causal relations among them. The study exploits a multivariate compact framework devised to favor the interpretation of changes of complexity in terms of modifications of the strength of the physiological links. The main features of this approach are the construction of a multivariate embedding space accounting for the interactions among HP, SAP and RESP variabilities, the optimization of the number of samples necessary to describe cardiovascular variability interactions (i.e. the embedding dimension), the estimation of the interaction delays between different variability series and between samples of the same series, the evaluation of the performance of two alternative classes of methods for the assessment of complexity and causality, i.e. the linear model-based (MB) and nonlinear model-free (MF) class, and the comparison between the two techniques most frequently utilized in the MF class, i.e. local predictability (LP) and conditional entropy (CE). The effect of senescence was assessed at baseline in supine resting condition (REST) and during sympathetic activation induced by changing posture from REST to active standing (STAND) in a cohort of 100 healthy subjects from 21 to 70 years subdivided into five bins of age each including 20 subjects.

## Methods

### General Definitions

Given *M* series *y_1_* = {*y_1_*(*n*), *n* = 1,…,*N*}, …, *y_M_* = {*y_M_*(*n*), *n* = 1,…,*N*} where *N* is the series length and *n* is the progressive counter, the series are first normalized to have zero mean and unit variance. We define *Ω* = {*y_1_*,…,*y_i_*,…,*y_j_*,…,*y_M_*} as the universe of the knowledge about the system under study. After labeling as ***Y*** *_j_^i^*(*n*) = [*y_j_*(*n*-*τ_j_^i^*),…,*y_j_*(*n*-*p_i_*)] with 1≤*i*,*j*≤*M*, the embedding vector formed by *p_j_^i^* = *p_i_*-*τ_j_^i^*+1 components of *y_j_*, where *τ_j_^i^* and *p_i_* represent the minimal and the maximal delay of influence of *y_j_* on *y_i_*, the multivariate embedding vector accounting for all signals present in *Ω* is ***Z*** *_i_*(*n*) = [***Y*** *_1_^i^*(*n*),…,***Y*** *_i_^i^*(*n*),…,***Y*** *_j_^i^*(*n*),…,***Y*** *_M_^i^*(*n*)] and has dimension
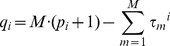
(1)where 1≤*τ_i_^i^*≤*p_i_* and 0≤*τ_m_^i^*≤*p_i_* with *m*≠*i*. The possibility that *τ_m_^i^* = 0 is introduced to account for instantaneous (i.e., non-delayed) effects from *y_m_* to *y_i_*. All the multivariate embedding vectors form a set ***Z*** *_i_* = {***Z*** *_i_*(*n*), *n* = *p_i_*+1,…,*N*}. These definitions can be easily extended to the universe *Ω* after the exclusion of *y_j_*, i.e. *Ω*\*y_j_* = {*y_1_*,…,*y_i_*,…,*y_M_*}. In this case the multivariate embedding vector obtained from ***Z*** *_i_*(*n*) after excluding ***Y*** *_j_^i^*(*n*) is ***Z*** *_i_*(*n*)\***Y*** *_j_^i^*(*n*) = [***Y*** *_1_^i^*(*n*),…,***Y*** *_i_^i^*(*n*),…,***Y*** *_M_^i^*(*n*)] and the set of all ***Z*** *_i_*(*n*)\***Y*** *_j_^i^*(*n*) is indicated as ***Z*** *_i_*\***Y*** *_j_^i^*.

### Linear MB Approach for the Assessment of Complexity

This technique computes complexity as the degree of unpredictability of *y_i_* in Ω according to a MB approach [25]. The multivariate linear regression model was exploited to describe the interactions of *y_i_* with all the signals present in *Ω* [25]. More specifically,
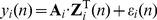
(2)


where ***A*** *_i_* is the *q_i_*x1 vector containing the coefficients of the model, ***A*** *_i_* = [***A*** *_1_^i^*,…,***A*** *_i_^i^*,…,***A*** *_j_^i^*,…,***A*** *_M_^i^*] with ***A*** *_j_^i^* = [*a_j_*(*τ_j_^i^*),…,*a_j_*(*p_i_*)], *p_i_* is the order of the model, the symbol ^T^ is the transpose operator, and *ε_i_* is the white noise with zero mean and variance *λ_i_^2^*. Equation (2) describes *y_i_*(*n*) as a linear combination of *y_j_*(*n*-*k*) with *τ_j_^i^*≤*k*≤*p_i_* and 1≤*j*≤*M* weighted by the constant coefficient *a_j_*(*k*) plus *ε_i_*(*n*) representing the part of *y_i_*(*n*) that cannot be predicted in *Ω*. We follow the criterion leading to the minimization of *λ_i_^2^* in Ω to identify ***A*** *_i_* [25]. Given the estimate of ***A*** *_i_*, 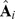, the prediction of *y_i_*(*n*) is
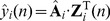
(3)


Defined the prediction error, *e_i_*(*n*), as the difference between *y_i_*(*n*) and its prediction, 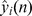, a measure of the unpredictability of *y_i_* in *Ω* is the mean square prediction error indicated as 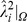 in the following. 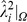 is computed as a function of the model order, *p_i_*, according to a procedure leading to add one sample for each signal present in *Ω* at every increment of *p_i_* (i.e. *M* samples at a time). Since at every increment of *p_i_* the in-sample ability of the model in fitting the data improves, 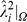 progressively decreases with *p_i_*, thus making practically useless the monitoring of 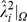 with *p_i_* to decide the optimal model order [25]. Therefore, instead of tracking 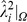 it is a common practice to monitor a figure of merit, here the Akaike figure of merit [26]. The Akaike figure of merit adds a term gradually increasing with *p_i_* to a function depending on 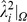, thus forcing the creation of a minimum. The model order at the minimum of the Akaike figure of merit will be indicated as p_i_^o^. 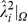 at p_i_^o^ is taken as a normalized complexity index (NCI) of *y_i_* in *Ω* based on the MB approach, and labeled as 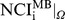. Unless necessary to stress the universe of knowledge, NCI_i_^MB^ will be adopted instead of 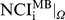 and, in this case, we will assume that the universe of knowledge is *Ω*. Since all the series are normalized to have unit variance, NCI_i_^MB^ ranges between 0 and 1, where 0 indicates null complexity of *y_i_* and its full predictability and 1 indicates maximal complexity of *y_i_* and its full unpredictability.

### Linear MB Approach for the Assessment of Causality

Causality from *y_j_* to *y_i_* based on MB approach is evaluated according to the causality ratio (CR)
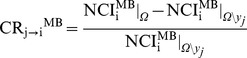
(4)


where 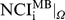 and 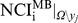 represent NCI_i_^MB^ assessed respectively in *Ω* and *Ω\y_j_*. CR_j→i_^MB^ quantifies the strength of the causal relation from *y_j_* to *y_i_* as the unpredictability decrement of *y_i_* due to the inclusion of *y_j_* in *Ω*\*y_j_*. 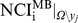 was computed by estimating the coefficients of the polynomials in *Ω\y_j_* while maintaining p_i_^o^ optimized in *Ω*. CR_j→i_^MB^<0 indicates that *y_j_* carries unique information about the future evolution of *y_i_* that cannot be derived from any signal in *Ω*\*y_j_*, and, according to the concept of Granger causality [12], it can be stated that *y_j_* Granger-causes *y_i_* in *Ω*.

### Nonlinear MF Approach for the Assessment of Complexity Based On LP

This technique computes complexity as the degree of unpredictability of *y_i_* in *Ω* [11]. The method hypothesizes that there is a function *f*(^.^) linking ***Z*** *_i_*(*n*) to *y_i_*(*n*). *y_i_*(*n*) is usually indicated as the image of ***Z*** *_i_*(*n*) through *f*(^.^) and, vice versa, ***Z*** *_i_*(*n*) is said to be the vector associated to *y_i_*(*n*) through *f*(^.^). The k-nearest-neighbor approach provides a local approximation of *f*(^.^) under smooth conditions and without making any specific assumption on the dynamical relationship linking ***Z*** *_i_*(*n*) to *y_i_*(*n*) [27], thus allowing the prediction of *y_i_*(*n*), 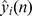, according to a MF approach. More specifically, 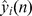 is computed in *Ω* as a combination over a subset of values of *y_i_* whose associated embedding vectors belong to the set of the *k* points closest to the embedding vector, ***Z*** *_i_*(*n*), associated to the value to be predicted, *y_i_*(*n*). Usually, the set of the closest points does not include vectors with time indexes close to *n* to reduce the correlation among the selected values of *y_i_* solely due to the temporal closeness of the associated embedding vectors [28]. To combine the *k* selected values of *y_i_*, the weighted mean, where the weights are the inverse of the distance from ***Z*** *_i_*(*n*) to each neighbor vector is a natural choice (i.e. zero-order predictor) [29]. The distance between vectors is calculated according to a predefined norm (here we use the maximum norm, i.e. the absolute value of the maximal difference between corresponding components) [30]. After calculating the prediction of *y_i_*, 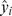, the square correlation coefficient between *y_i_* and 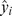, *r_i_* ^2^, can be assessed [31]. *r_i_* ^2^ is bounded between 0 and 1, respectively indicating null and perfect predictability of *y_i_*. Predictability depends on the construction of the multivariate embedding space. Here we exploit a strategy growing up progressively the embedding space by adding one lagged component at a time selected according to a given criterion [14], [32]. Let us refer to as candidate the lagged component being possibly selected as a new coordinate of the embedding space. Initially (i.e. when *q_i_* = 0), the set of candidates tested to predict *y_i_*(*n*) is {*y*_1_(*n-τ_1_^i^*),…,*y*_1_(*n*-*p_i_*),…,*y_i_*(*n*-*τ_i_^i^*),…,*y_i_*(*n*-*p_i_*),…,*y_j_*(*n-τ_j_^i^*),…,*y_j_*(*n*-*p_i_*),…,*y_M_*(*n-τ_M_^i^*),…,*y_M_*(*n*-*p_i_*)}. All candidates are tested by assessing *r_i_^2^* and the new component is selected as the one maximizing *r_i_^2^*. Then, the selected component is retained, *q_i_* is increased by 1 and the set of candidates is reduced by excluding samples of a signal with time indexes more recent or equal to any component of the same signal already exploited to form the multivariate embedding space, thus avoiding duplicate selections and speeding up reconstruction. The process continues until the set of candidates is empty. *r_i_^2^* varies with *q_i_* and, provided that *Ω* is helpful to predict *y_i_*, its course is the result of two opposite tendencies: i) a larger number of components increases the ability of ***Z*** *_i_* to predict *y_i_* by allowing a more accurate unfolding of the trajectories in the multivariate embedding space and reducing the ambiguities in mapping ***Z*** *_i_* to future samples, thus leading to an increase of *r_i_^2^* with *q_i_*; ii) in presence of noise and time series of limited length, vectors in ***Z*** *_i_* progressively take apart with *q_i_* in the multivariate embedding space and, consequently, the ability of the *k* closest points to predict *y_i_* worsens, thus leading to a decrease of *r_i_^2^* with *q_i_*. Therefore, *r_i_^2^* exhibits a maximum over *q_i_*,or a minimum if the functional 1-*r_i_^2^* is considered [31]. Conversely, if *y_i_* cannot be predicted in *Ω, r_i_^2^* remains close to 0 regardless of *q_i_*. The largest value of *r_i_^2^* over *q_i_*, max(*r_i_^2^*), is complemented to 1 (i.e. 1-max(*r_i_^2^*)) to obtain the NCI of *y_i_* based on LP in *Ω* labeled as 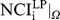. Unless necessary to stress the universe of knowledge, from now on NCI_i_^LP^ will be adopted instead of 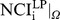 and, in this case, we will assume that the universe of knowledge is *Ω*. NCI_i_^LP^ ranges from 0, indicating null complexity and perfect predictability of *y_i_*, to 1, representing maximal complexity and full unpredictability of *y_i_*. *q_i_* at NCI_i_^LP^, q_i_^LPo^, represents the optimal number of components of ***Z*** *_i_*(*n*) leading to the best prediction of *y_i_*(*n*). q_i_^LPo^ is bounded between 0 and *q_i_* given in (1).

### Nonlinear MF Approach for the Assessment of Causality Based on LP

Causality from *y_j_* to *y_i_* based on LP is evaluated according to the CR
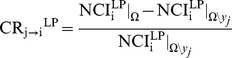
(5)


where 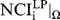 and 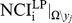 represent NCI_i_^LP^ assessed respectively in *Ω* and *Ω\y_j_*. CR_j→i_^LP^ quantifies the strength of the causal relation from *y_j_* to *y_i_* as the decrement of uncorrelation between *y_i_* and 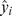 due to the inclusion of *y_j_* in *Ω*\*y_j_*. In the assessment of CR_j→i_^LP^, 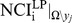 was computed by excluding the components of *y_j_* from the optimal embedding vector leading to the calculation of 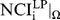. With this expedient, if the optimal embedding vector does not contain any component of *y_j_*, CR_j→i_^LP^ = 0, indicating the absence of causality from *y_j_* from *y_i_*. Conversely, CR_j→i_^LP^<0 indicates that *y_j_* carries unique information about the future evolution of *y_i_* that cannot be derived from any signal in *Ω*\*y_j_*, and, according to the concept of Granger causality [12], it can be stated that *y_j_* Granger-causes *y_i_* in *Ω*.

### Nonlinear MF Approach for the Assessment of Complexity Based on CE

The technique computes complexity as the amount of information carried by *y_i_* that cannot be derived from any of the series present in *Ω* through the calculation of the CE [33]. A k-nearest-neighbor approach can be exploited to compute the CE of *y_i_* in *Ω* [34]. CE is computed as the average value of the Shannon entropy of the conditional distribution of *y_i_*(*n*) given ***Z*** *_i_*(*n*). The distribution of *y_i_*(*n*) given ***Z*** *_i_*(*n*) is built by considering the subset of values of *y_i_* whose associated embedding vectors belong to the set of the *k* points closest to ***Z*** *_i_*(*n*). The Shannon entropy of the distribution of *y_i_*(*n*) given ***Z*** *_i_*(*n*) is calculated as the negative natural logarithm of the average probability that two samples of the distribution of *y_i_*(*n*) given ***Z*** *_i_*(*n*) are at distance closer than *ε* [34]. In this study the tolerance *ε* was set to 10% of the difference between the 84^th^ and the 16^th^ percentile of *y_i_* and the distance was computed, as in the LP approach, using the maximum norm. CE is bounded between 0 and the Shannon entropy of *y_i_* indicating respectively the minimal and the maximal amount of information contained in *y_i_*. CE is smaller than the Shannon entropy of *y_i_* when the set of conditioning vectors, ***Z*** *_i_*, is helpful to reduce the uncertainty associated to *y_i_*. CE depends on the construction of the multivariate embedding space. Here we exploit a strategy to construct and optimize the embedding space analogous to the one exploited by the LP approach. Only the optimization criterion is different. Indeed, the new component is selected among the set of candidates as the one producing the maximal decrement of the uncertainty of *y_i_* (i.e. the minimum of CE) compared to the maximal level of uncertainty of *y_i_* quantified by the Shannon entropy of *y_i_* [14]. CE varies with q_i_ and, provided that *Ω* is helpful to reduce the uncertainty of *y_i_*, the course of CE is the result of two opposite tendencies: i) a larger number of components increases the ability of ***Z*** *_i_* to reduce the uncertainty of *y_i_* because longer conditioning patterns have better possibilities in fixing future samples, thus leading to a decrease of CE with *q_i_*; ii) in presence of noise and time series of limited length, vectors in ***Z*** *_i_* progressively take apart with *q_i_* in the multivariate embedding space and, consequently, the ability of the *k* closest points to limit uncertainty vanishes, thus leading to the increase of CE with *q_i_*. Therefore, CE exhibits a minimum over *q_i_*. Conversely, if the information carried by *y_i_* cannot be reduced in *Ω*, CE remains close to the Shannon entropy of *y_i_* regardless of *q_i_*. The minimum of the CE over *q_i_* is normalized by the Shannon entropy of *y_i_* to obtain a NCI of *y_i_* based on CE in *Ω*, labeled as 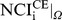. Unless necessary to stress the universe of knowledge, from now on NCI_i_^CE^ will be adopted instead of 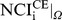 and, in this case, we assume that the universe of knowledge is *Ω*. NCI_i_^CE^ ranges from 0, indicating null information and complexity of *y_i_*, to 1, representing maximal information and complexity of *y_i_*. *q_i_* at NCI_i_^CE^, q_i_^CEo^, represents the optimal number of components of ***Z*** *_i_*(*n*) leading to the maximal reduction of uncertainty of *y_i_*. q_i_^CEo^ is bounded between 0 and *q_i_* given in (1).

### Nonlinear MF Approach for the Assessment of Causality Based on CE

Causality from *y_j_* to *y_i_* based on CE is computed according to the CR
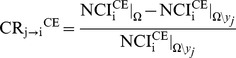
(6)


where 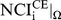 and 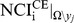 represent NCI_i_^CE^ assessed respectively in *Ω* and *Ω\y_j_*. CR_j→i_^CE^ quantifies the strength of the causal link from *y_j_* to *y_i_* as the decrement of information carried by *y_i_* attributable solely to the inclusion of *y_j_* in *Ω\y_j_*. We use the same expedient exploited in the calculation of CR_j→i_^LP^ resulting in CR_j→i_^CE^ = 0 when no components of *y_j_* are present in the optimal embedding vector leading to the computation of 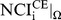. Conversely, CR_j→i_^CE^<0 indicates that *y_j_* is capable to reduce the uncertainty of *y_i_* to a level that cannot be achieved by exploiting any signal in *Ω*\*y_j_* and, according to the concept of transfer entropy [35], it can be stated that *y_j_* causes *y_i_* in *Ω*.

## Experimental Protocol and Data Analysis

### Ethics Statement

The study was performed according to the Declaration of Helsinki and it was approved by the Human Research Ethics Committee of the Federal University of São Carlos (protocol number 173/2011). A written informed consent was obtained from all subjects.

### Experimental Protocol

We studied 100 nonsmoking healthy humans (54 males, age from 21 to 70 years, median = 45 years; weight from 43 to 100 Kg, median = 71 Kg; height from 146 to 197 cm, median = 167 cm; body mass index (BMI) from 17.4 to 33.4 Kg^.^m^−2^, median = 25 Kg^.^m^−2^). The population was composed by 20 subjects in each of the following bins: from 21 to 30 years (10 males, median age = 26 years, median weight = 71 Kg, median height = 168 cm, median BMI = 23.9 Kg^.^m^−2^); from 31 to 40 years (11 males, median age = 34 years, median weight = 69 Kg, median height = 168 cm, median BMI = 24.8 Kg^.^m^−2^); from 41 to 50 years (10 males, median age = 45 years, median weight = 70 Kg, median height = 167 cm, median BMI = 25.4 Kg^.^m^−2^); from 51 to 60 years (10 males, median age = 55 years, median weight = 71 Kg, median height = 169 cm, median BMI = 25.1 Kg^.^m^−2^); from 61 to 70 years (13 males, median age = 65 years, median weight = 72 Kg, median height = 164 cm, median BMI = 26.7 Kg^.^m^−2^). The population was balanced in terms of gender to limit the influences of this confounding factor on the analysis [36]. The peak oxygen uptake, peak 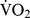, was computed during a maximum cardiopulmonary exercise test performed on a treadmill using a ramp protocol. The criteria for exercise interruption were based on [37] and the assessment was made according to [38]. In our population the median (25^th^–75^th^ percentile) of the peak 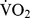 in each age bin was 2271 (1574–2894), 2340 (1993–3135), 2384 (1712–3080), 2026 (1363–2484) and 1825 (1436–2025) mL^.^min^−1^. All the subjects were apparently healthy, had no history and no clinical evidence of any disease based on clinical and physical examinations, laboratory tests, standard electrocardiogram (ECG) and on a maximum cardiopulmonary exercise test conducted by a physician. They were not taking any medication known to interfere with cardiovascular control. Smokers and habitual drinkers were excluded from this study. All subjects were evaluated in the afternoon. The experiments were carried out in a climatically controlled room (22–23°C) with relative air humidity at 40–60%. Subjects were instructed not to consume caffeinated and alcoholic beverages as well as not to perform strenuous exercises on the day before the recording. They were also instructed to ingest a light meal at least 2 hours prior to the test. On the experimental day, the subjects were interviewed and examined before the test to verify whether they were in good health and they had a regular night sleep. Prior to the recording, the volunteers were made familiar with the equipment and with the experimental procedure. During the entire protocol the subjects breathed spontaneously and they were not allowed to talk.

ECG (modified lead I), continuous plethysmographic arterial pressure (Finometer PRO, Finapress Medical System, The Netherlands) and respiratory movements via thoracic belt (Marazza, Monza, Italy) were digitalized using a commercial device (BioAmp Power Lab, AD Instruments, Australia). Signals were sampled at 400 Hz. The arterial pressure was measured from the middle finger of the left hand being maintained at the level of heart by fixing the subject’s arm to his thorax. All the experimental sessions of the protocol included two periods in the same order: 1) 15 minutes at REST; 2) 15 minutes during STAND. Before REST we allowed 10 minutes for stabilization. The arterial pressure signal was cross-calibrated in each session using a measure provided by a sphygmomanometer at the onset of REST. The auto-calibration procedure of the arterial pressure device was switched off after the first automatic calibration at the onset of the session. Analyses were performed after about 2 minutes from the start of each period.

### Extraction of the Beat-to-beat Variability Series

After detecting the R-wave on the ECG and locating its peak using parabolic interpolation, HP was approximated as the temporal distance between two consecutive parabolic apexes. The maximum of arterial pressure inside of the *n*-th HP, HP(*n*), was taken as the *n*-th SAP, SAP(*n*). The signal of the thoracic movements was down-sampled once per cardiac beat at the occurrence of the first R-wave peak delimiting HP(*n*), thus obtaining the *n*-th RESP measure, RESP(*n*). HP(*n*), SAP(*n*) and RESP(*n*) were expressed in ms, mmHg and arbitrary units (a.u.) respectively. The occurrences of R-wave and SAP peaks were carefully checked to avoid erroneous detections or missed beats. After extracting the series HP = {HP(*n*), *n* = 1,…,*N*}, SAP = {SAP(*n*), *n* = 1,…,*N*} and RESP = {RESP(*n*), *n* = 1,…,*N*}, where *n* is the progressive cardiac beat counter and *N* is the total cardiac beat number, sequences of 256 consecutive measures were randomly selected inside REST and STAND periods, thus focusing short-term cardiovascular regulatory mechanisms [3]. If evident nonstationarities, such as very slow drifting of the mean or sudden changes of the variance, were present in spite of the linear detrending, the random selection was carried out again.

### Traditional Index Calculation

Traditional time domain parameters such as mean and variance of HP and SAP were calculated and indicated as µ_HP_, µ_SAP_, σ^2^_HP_ and σ^2^_SAP_. They were expressed in ms, mmHg, ms^2^ and mmHg^2^ respectively. We assessed cardiac baroreflex sensitivity (BRS) according the baroreflex sequence method [39]. The technique relies on the search for sequences characterized by the contemporaneous increase (positive sequence) or decrease (negative sequence) of HP and SAP. Both positive and negative sequences are referred to as baroreflex sequences. They are identified according to the following prerequisites: 1) the length of the sequences was four beats (three increases or decreases); 2) the lag between HP and SAP values was set to 0; 3) the total HP variation was larger than 5 ms; 4) the total SAP variation was larger than 1 mmHg; v) the correlation coefficient in the plane [SAP(*n*),HP(*n*)] was larger than 0.85. When a baroreflex sequence matched with the above mentioned prerequisites the slope of the regression line in the plane [SAP(*n*),HP(*n*)] was calculated and averaged over all baroreflex sequences. This average was indicated hereafter as BRS and expressed as ms^.^mmHg^−1^. The total number of baroreflex sequences was also retained and divided by the number of meaningful SAP ramps where a SAP ramp was identified as a sequence of three consecutive SAP increases or decreases leading to a total SAP variation larger than 1 mmHg and a correlation coefficient in the plane [*n*,SAP(*n*)] larger than 0.85. This index was referred to as cardiac baroreflex effectiveness index (BEI) [40] and expressed as dimensionless units. This index can be considered an index of causality from SAP to HP because it gives the proportion of SAP ramps capable of evoking a meaningful cardiac baroreflex response.

### Complexity and Causality Indexes Calculation

NCI was calculated from HP, SAP and RESP series (i.e. NCI_HP_, NCI_SAP_ and NCI_RESP_) according to MB, LP and CE approaches. CR was computed from any pair of series (i.e. from SAP and RESP to HP, CR_SAP→HP_ and CR_RESP→HP,_ from HP and RESP to SAP, CR_HP→SAP_ and CR_RESP→SAP,_ from HP and SAP to RESP, CR_HP→RESP_ and CR_SAP→RESP_) according to MB, LP and CE approaches. In the MB approach the optimal model order was chosen as the one minimizing the Akaike figure of merit in the range from 1 to 8. In the case of LP and CE techniques the set of initial candidates was formed by 8 components for each series. The delays τ_SAP_^HP^ and τ_RESP_^HP^ were set to 0 to allow the description of the fast vagal reflex (within the same cardiac beat) capable to modify HP in response to changes of SAP and RESP [41], [42]. The delay τ_RESP_^SAP^ was set to 0 to account for the potential rapid effect of RESP on SAP due to the immediate transfer of an alteration of intrathoracic pressure on SAP value [1]. The delay τ_HP_^SAP^ was set to 1 due to the measurement conventions preventing that the modification of HP(*n*) can affect SAP(*n*) [18]. According to [43] actions of HP and SAP on RESP were slower (i.e. they cannot occur in the same beat), thus leading to τ_HP_^RESP^ = 1 and τ_SAP_^RESP^ = 1. The number of nearest neighbors, *k*, was set to 30 for both LP and CE approaches [31], [34].

### Statistical Analysis

Two way repeated measures analysis of variance (one factor repetition, Holm-Sidak test for multiple comparisons) was utilized to test the significance of the differences between the optimal model order assessed according to the MB approach and the number of components utilized to build the optimal multivariate embedding space according to LP and CE methods within the experimental condition (i.e. REST or STAND) while varying the method (i.e. MB, LP and CE) and within the method while varying the experimental condition. The null hypothesis of Normal distribution of all series and of all parameters extracted from them was tested according to Kolmogorov-Smirnov test. Linear regression analysis of µ_HP_, σ^2^_HP_, µ_SAP,_ σ^2^_SAP_, BRS and BEI on age was carried out. If Normality test over µ_HP_, σ^2^_HP_, µ_SAP,_ σ^2^_SAP_, BRS and BEI passed Pearson product moment correlation coefficient was calculated. Otherwise, Spearman rank order correlation coefficient was computed. The same procedure was carried out to check the dependence of NCI and CR on age. Statistical analysis was carried out using a commercial statistical program (Sigmastat, SPSS, ver.3.0.1). A p<0.05 was always considered as significant.

## Methodological Results

### Comparison of the MB, LP and CE Approaches

**Figure 1:**
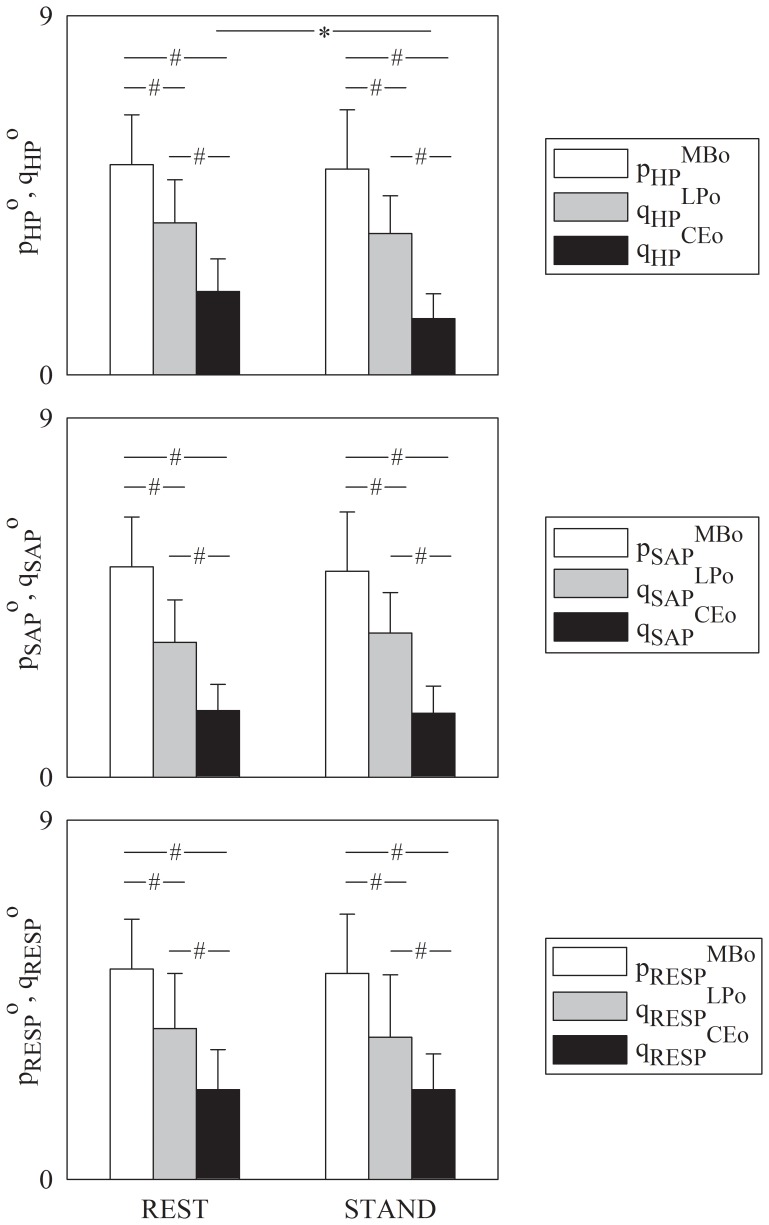
Comparison of the MB, LP and CE approaches. Grouped bar-graphs show the mean (plus standard deviation) of the optimal model order, p^o^, leading to the minimum of the Akaike figure of merit according to the MB approach (white bar) and of the number of components, q^o^, utilized to build the optimal multivariate embedding space leading to smallest unpredictability according to the LP method (gray bar) and to the minimal amount of information according to the CE technique (black bar). p^o^ and q^o^ are shown as a function of the experimental condition (i.e. REST and STAND) and values are pooled together regardless of age. The designated effect series are HP, SAP and RESP in (a), (b) and (c) respectively. The symbols ^#^ indicates a significant difference with p<0.05 within the same experimental condition (i.e. REST or STAND) while varying the method. The symbols ^*^ indicates a significant difference with p<0.05 within the method while varying the experimental condition.

Figure 1 shows the grouped bar-graphs of the optimal model order, p^o^, according to the minimum of the Akaike figure of merit derived from the MB approach (white bar) and the number of components utilized to build the optimal embedding space, q^o^, according to the smallest unpredictability obtained from the LP technique (gray bar), and to minimal amount of information derived from the CE method (black bar). Values of p^o^ and q^o^ are plotted as mean plus standard deviation over the entire cohort of subjects regardless of age and as a function of the experimental condition (i.e. REST and STAND). p^o^ and q^o^ were assessed while varying the designated effect series, i.e. HP, SAP and RESP in Figs. 1a,b,c respectively. Independently of the assigned effect series methods provided significantly different values of p^o^ and q^o^ within the same experimental condition (i.e. REST or STAND). These differences indicated that the optimal description of the interactions among variability series was achieved using the largest and the smallest number of components by the MB and CE approaches respectively, while the LP technique was in between them. It is worth noting that no difference between experimental conditions within the same method was observable except in the case of q_HP_^CEo^ (Fig. 1a): q_HP_^CEo^ during STAND was significantly smaller than q_HP_^CEo^ at REST.

## Experimental Results

### Linear Regression Analysis of Traditional Parameters on Age

Correlation analysis was carried out to assess the association of traditional parameters derived from HP and SAP variability on age at REST. While µ_HP_ and BEI was unrelated to age, σ^2^_HP_, µ_SAP_, σ^2^_SAP_ and BRS were found significantly correlated with age. The correlation coefficient was negative in the case of σ^2^_HP_ and BRS, thus indicating that σ^2^_HP_ and BRS progressively decreased with age, and it was positive in the case of µ_SAP_ and σ^2^_SAP_, thus evidencing that µ_SAP_ and σ^2^_SAP_ increased with age. Remarkably, the probability of the type I error assessed over σ^2^_HP_, σ^2^_SAP_ and BRS were at least three orders of magnitude smaller than that relevant to µ_SAP_.

Correlation analysis was carried out to assess the association of traditional parameters obtained from HP and SAP variability on age during STAND as well. At difference with REST µ_HP_ and BEI was significantly linearly correlated with age, while no linear relation was detected between σ^2^_SAP_ and age. The sign of the correlation coefficient of µ_HP_ and BEI suggests that the orthostatic challenge induced a tachycardic response and an association from SAP to HP becoming less and less important with age. Similarly to REST the progressive decrease of σ^2^_HP_ and BRS and the gradual increase of µ_SAP_ was significant.

### Linear Regression Analysis of Complexity Indexes on Age

Table 1 reports the results of the linear regression analysis of complexity parameters on age at REST. Independently of the approach (i.e. MB, LP or CE) NCI_HP_ was significantly linearly correlated with age. The correlation coefficients of NCI_HP_^MB^, NCI_HP_^LP^ and NCI_HP_^CE^ were negative, thus suggesting a progressive loss of complexity of HP dynamics with age. The analysis of SAP complexity points out differences among the methods. Indeed, while NCI_SAP_ derived from MF techniques, NCI_SAP_^LP^ and NCI_SAP_^CE^, were significantly associated with age, NCI_SAP_^MB^ was unrelated to it. The correlation coefficients of NCI_SAP_^LP^ and NCI_SAP_^CE^ were negative, thus indicating a progressive loss of complexity of the SAP series with age. Regardless of the method complexity of RESP dynamics was unaffected by aging.

**Table 1:** Linear regression analysis of the complexity parameters on age at REST.

	r	significance
NCI_HP_^MB^	−0.350	Yes
NCI_HP_^LP^	−0.348	Yes
NCI_HP_^CE^	−0.317	Yes
NCI_SAP_^MB^	−0.0989	No
NCI_SAP_^LP^	−0.264	Yes
NCI_SAP_^CE^	−0.243	Yes
NCI_RESP_^MB^	0.0022	No
NCI_RESP_^LP^	0.0572	No
NCI_RESP_^CE^	0.129	No

NCI_HP_^MB^, NCI_HP_^LP^, NCI_HP_^CE^ = normalized complexity index of HP series derived from MB, LP and CE approaches respectively; NCI_SAP_^MB^, NCI_SAP_^LP^, NCI_SAP_^CE^ = normalized complexity index of SAP series derived from MB, LP and CE approaches respectively; NCI_RESP_^MB^, NCI_RESP_^LP^, NCI_RESP_^CE^ = normalized complexity index of RESP series derived from MB, LP and CE approaches respectively; r = Pearson product-moment or Spearman rank order correlation coefficient; Yes/No = the variable is/is not significantly related to age with p<0.05.

Table 2 reports the results of the linear regression analysis of complexity indexes on age during STAND. In this condition solely the complexity of HP series was influenced by senescence. Indeed, NCI_SAP_ and NCI_RESP_ were unrelated to age. NCI_HP_ was found significantly related to age when computed according to MB and LP approaches, while NCI_HP_^CE^ was independent of it. The correlation coefficients of NCI_HP_^MB^ and NCI_HP_^LP^ on age were positive, thus suggesting that HP variability during STAND became more and more unpredictable with age.

**Table 2:** Linear regression analysis of the complexity parameters on age during STAND.

	r	significance
NCI_HP_^MB^	0.214	Yes
NCI_HP_^LP^	0.208	Yes
NCI_HP_^CE^	0.179	No
NCI_SAP_^MB^	0.086	No
NCI_SAP_^LP^	−0.0538	No
NCI_SAP_^CE^	−0.0361	No
NCI_RESP_^MB^	−0.0424	No
NCI_RESP_^LP^	−0.0570	No
NCI_RESP_^CE^	−0.0564	No

NCI_HP_^MB^, NCI_HP_^LP^, NCI_HP_^CE^ = normalized complexity index of HP series derived from MB, LP and CE approaches respectively; NCI_SAP_^MB^, NCI_SAP_^LP^, NCI_SAP_^CE^ = normalized complexity index of SAP series derived from MB, LP and CE approaches respectively; NCI_RESP_^MB^, NCI_RESP_^LP^, NCI_RESP_^CE^ = normalized complexity index of RESP series derived from MB, LP and CE approaches respectively; r = Pearson product-moment or Spearman rank order correlation coefficient; Yes/No = the variable is/is not significantly related to age with p<0.05.

### Linear Regression Analysis of Causality Indexes on Age

Table 3 reports the results of the linear regression analysis of causality parameters on age at REST. Regardless of the method (i.e. MB, LP and CE) CR_SAP→HP_, CR_RESP→SAP_, CR_HP→RESP_ and CR_SAP→RESP_ showed the same association with age: while CR_SAP→HP_, CR_HP→RESP_ and CR_SAP→RESP_ were unaffected by age, CR_RESP→SAP_ was linearly associated to it. The correlation coefficient of CR_RESP→SAP_ was positive, thus suggesting that aging reduced the strength of the causal link from RESP to SAP. The methods were not in full agreement in the case of CR_HP→SAP_ and CR_RESP→HP_. Indeed, the MB approach was the unique technique detecting a significant linear relation of CR_HP→SAP_ on age. The correlation coefficient of CR_HP→SAP_^MB^ was positive, thus indicating that the strength of the causal link from HP to SAP diminished with age. In the case of CR_RESP→HP_, both LP and CE approaches found a significant linear association with age, while the MB one was unable to detect it. The correlation coefficients of CR_RESP→HP_^LP^ and CR_RESP→HP_^CE^ were positive, thus suggesting that the influence of RESP on HP became more and more ineffective with age.

**Table 3:** Linear regression analysis of the causality parameters on age at REST.

	r	significance
CR_SAP→HP_^MB^	0.0864	No
CR_SAP→HP_^LP^	−0.0676	No
CR_SAP→HP_^CE^	−0.0514	No
CR_HP→SAP_^MB^	0.287	Yes
CR_HP→SAP_^LP^	0.0233	No
CR_HP→SAP_^CE^	0.104	No
CR_RESP→HP_^MB^	−0.0493	No
CR_RESP→HP_^LP^	0.201	Yes
CR_RESP→HP_^CE^	0.361	Yes
CR_RESP→SAP_^MB^	0.249	Yes
CR_RESP→SAP_^LP^	0.229	Yes
CR_RESP→SAP_^CE^	0.259	Yes
CR_HP→RESP_^MB^	0.119	No
CR_HP→RESP_^LP^	0.173	No
CR_HP→RESP_^CE^	−0.0399	No
CR_SAP→RESP_^MB^	−0.128	No
CR_SAP→RESP_^LP^	−0.180	No
CR_SAP→RESP_^CE^	0.0978	No

CR_SAP→HP_^MB^, CR_SAP→HP_^LP^, CR_SAP→HP_^CE^ = causality ratio from SAP to HP series derived from MB, LP and CE approaches; CR_HP→SAP_^MB^, CR_HP→SAP_^LP^, CR_HP→SAP_^CE^ = causality ratio from HP to SAP series derived from MB, LP and CE approaches; CR_RESP→HP_^MB^, CR_RESP→HP_^LP^ CR_RESP→HP_^CE^ = causality ratio from RESP to HP series derived from MB, LP and CE approaches; CR_RESP→SAP_^MB^, CR_RESP→SAP_^LP^ CR_RESP→SAP_^CE^ = causality ratio from RESP to SAP series derived from MB, LP and CE approaches; CR_HP→RESP_^MB^, CR_HP→RESP_^LP^, CR_HP→RESP_^CE^ = causality ratio from HP to RESP series derived from MB, LP and CE approaches; CR_SAP→RESP_^MB^, CR_SAP→RESP_^LP^ CR_SAP→RESP_^CE^ = causality ratio from SAP to RESP series derived from MB, LP and CE approaches; r = Pearson product-moment or Spearman rank order correlation coefficient; Yes/No = the variable is/is not significantly related to age with p<0.05.

Table 4 reports the results of the linear regression analysis of causality indexes on age during STAND. The association of CR_RESP→HP_, CR_HP→RESP_ and CR_SAP→RESP_ with age did not depend on the method (i.e. MB, LB and CE). Indeed, all the three approaches found that CR_RESP→HP_, CR_HP→RESP_ and CR_SAP→RESP_ were unrelated to age. Conversely, the association of CR_SAP→HP_, CR_HP→SAP_ and CR_RESP→SAP_ with age depended on the method. Only the LP approach was able to detect the significant linear relation of CR_SAP→HP_ with age. The correlation coefficient of CR_SAP→HP_^LP^ was positive, thus indicating that the strength of the causal link from SAP to HP became weaker and weaker with age. The significant linear association of CR_HP→SAP_ with age was detected only by the MB technique. The correlation coefficient of CR_HP→SAP_^MB^ was positive, thus indicating that a progressive decrease of the intensity of the causal relation from HP to SAP with age. In the case of CR_RESP→SAP_, only the CE method was unable to detect a significant linear relation of the strength of the causal link from RESP to SAP with age. Indeed, the correlation coefficients of CR_RESP→SAP_^MB^ and CR_RESP→SAP_^LP^ were significant and positive, thus suggesting that the senescence reduced the association between RESP and SAP variability in the temporal direction from RESP to SAP.

**Table 4:** Linear regression analysis of the causality parameters on age during STAND.

	r	significance
CR_SAP→HP_^MB^	0.180	No
CR_SAP→HP_^LP^	0.316	Yes
CR_SAP→HP_^CE^	−0.0107	No
CR_HP→SAP_^MB^	0.255	Yes
CR_HP→SAP_^LP^	−0.0742	No
CR_HP→SAP_^CE^	0.0387	No
CR_RESP→HP_^MB^	−0.00073	No
CR_RESP→HP_^LP^	−0.154	No
CR_RESP→HP_^CE^	−0.152	No
CR_RESP→SAP_^MB^	0.243	Yes
CR_RESP→SAP_^LP^	0.219	Yes
CR_RESP→SAP_^CE^	0.0755	No
CR_HP→RESP_^MB^	0.0103	No
CR_HP→RESP_^LP^	−0.0107	No
CR_HP→RESP_^CE^	−0.123	No
CR_SAP→RESP_^MB^	0.185	No
CR_SAP→RESP_^LP^	−0.064	No
CR_SAP→RESP_^CE^	0.0239	No

CR_SAP→HP_^MB^, CR_SAP→HP_^LP^, CR_SAP→HP_^CE^ = causality ratio from SAP to HP series derived from MB, LP and CE approaches; CR_HP→SAP_^MB^, CR_HP→SAP_^LP^, CR_HP→SAP_^CE^ = causality ratio from HP to SAP series derived from MB, LP and CE approaches; CR_RESP→HP_^MB^, CR_RESP→HP_^LP^ CR_RESP→HP_^CE^ = causality ratio from RESP to HP series derived from MB, LP and CE approaches; CR_RESP→SAP_^MB^, CR_RESP→SAP_^LP^ CR_RESP→SAP_^CE^ = causality ratio from RESP to SAP series derived from MB, LP and CE approaches; CR_HP→RESP_^MB^, CR_HP→RESP_^LP^, CR_HP→RESP_^CE^ = causality ratio from HP to RESP series derived from MB, LP and CE approaches; CR_SAP→RESP_^MB^, CR_SAP→RESP_^LP^ CR_SAP→RESP_^CE^ = causality ratio from SAP to RESP series derived from MB, LP and CE approaches; r = Pearson product-moment or Spearman rank order correlation coefficient; Yes/No = the variable is/is not significantly related to age with p<0.05.

## Discussion on the Methodological Findings

The methodological findings of this study can be summarized as follows: i) the study proposes a compact framework for the assessment of complexity of cardiovascular variability and causality of their interactions in a multivariate embedding space; ii) the common feature of all techniques designed within the proposed framework is the optimization of the embedding dimension; iii) this framework facilitates the comparison between linear MB and nonlinear MF classes and, inside the MF class, between LF and CE techniques; iv) the simultaneous assessment of complexity and causality favors the interpretation of changes of complexity in terms of modifications of the strength of physiological relations.

### A Multivariate Compact Framework for the Assessment of Complexity and Causality in Cardiovascular Variability Series

We applied a multivariate compact framework for the assessment of dynamical complexity and causality over an arbitrary set of signals, *Ω*. In the case of MB and LP approaches the complexity of an assigned effect series was evaluated as its degree of unpredictability in *Ω* (i.e. the portion of normalized variance of the assigned effect series that cannot be explained based on all signals present in *Ω*). In the case of the CE method the complexity of the selected effect series was assessed as the amount of information carried by the designated effect series that cannot be derived from the signals present in *Ω*. In the case of MB and LP approaches the strength of the causal relation from a cause series to an effect one in *Ω* was computed, according to the notion of Granger causality [12], as the fractional decrement of unpredictability of the assigned effect series resulting from the inclusion of the cause series into the incomplete *Ω* disregarding the supposed cause. In the case of CE method causality was assessed, according to the notion of information transfer [35], as the fractional decrement of the information carried by the selected effect series resulting from the inclusion of the cause series into the incomplete set of conditioning signals formed by *Ω* devoid of the presumed cause.

One of the main features of the framework is the assessment of complexity of a designated effect series into a multivariate embedding space built in *Ω*. This characteristic distinguishes the proposed approach from univariate methods exploiting embedding spaces built over a unique series [29], [30], [44]–[47]. In our application the multivariate embedding space might provide a more efficient description of the overall complexity of the cardiovascular control than the univariate embedding space usually constructed using solely the HP components because it allows a more complete representation of the dynamical behavior of the subsystems contributing to the overall functioning of the cardiovascular system. Indeed, the joint exploitation of variables directly linked to cardiac, vascular and respiratory subsystems (i.e. HP, SAP and RESP) might reveal portions of the cardiovascular system that otherwise might remain unveiled or undervalued using the time course of a single variable. Since the multivariate embedding space is the starting point for causality analysis [12], [48], the proposed framework allows the joint evaluation of complexity and causality indexes. At difference with most of the applications of causality approaches, the proposed framework is grounded on the optimization of the multivariate embedding dimension, thus avoiding the common practice of its arbitrary assignment [13], [49], [50] or its opportunistic setting to a value allowing the best separation among populations and/or experimental conditions [16]. In our study the multivariate embedding dimension was optimized by MB, LP and CE approaches according to, respectively, the classical Akaike information criterion, the maximization of the predictability of the assigned effect series, and the minimization of the information carried by the assigned effect series. The optimization of the multivariate embedding dimension allows the reduction of the number of parameters needed to be set, thus favoring the automatic computation of complexity and causality markers.

Another element of novelty of the proposed framework is the possibility of performing the direct comparison between MB and MF classes of methods. The MB methods interpret the dynamical interactions according to a model, while the MF ones are completely data-driven and do not impose any particular predefined structure to the dynamics. While several studies advocated the use of MF approaches for the computation of causality due to their straight adherence to the data and their ability to account for nonlinearities of any type [16], [48], the presumed superiority of MF tools was never checked over real, short and noisy data via a direct comparison to MB methods. Understanding the additional information that might be derived from MF methods compared to MB ones is a relevant issue because MB approaches, especially if based on multivariate linear regression models, are well-established, robust and computationally efficient [25]. The proposed framework compares MF approaches with a traditional MB method based on multivariate linear regression model [25]. In this MB approach the multivariate embedding space was built sequentially by adding *M* delayed components at a time, one for any of the signals present in *Ω*. This strategy, quite common in applications of multivariate linear regression models [13], [51]–[55], might lead to an overparametrization of the model and to possible suboptimality of the description of the interactions among signals because the number of components exploited in any regression is constrained to be the same. Conversely, the strategy followed by both the proposed MF approaches (i.e. LP and CE) leads to a reconstruction of the multivariate embedding space using fewer components. Defined as parsimoniousness of a model its ability to describe interactions among signals using a small number of components, it can be stated that the proposed MF approaches are more parsimonious than the MB one. Indeed, at every increment of the embedding dimension we tested all the candidate components and we selected the one providing the maximal correlation between the original effect series and its prediction in the case of the LP approach, and the maximal reduction of uncertainty about the future evolution of the effect series in the case of the CE method. Parsimoniousness is a quality because limits overfitting even though, when it is excessive, it might become a disadvantage because it leads to underfitting. The optimization of the embedding dimension allows the best unfolding of the system dynamics as well as it solves the issue of setting the delay of the interactions from any cause signal to the effect one and the lag between components belonging to the same signal. The setting of these parameters is a non trivial issue. Indeed, the interaction delay is usually set according to physiological considerations about the latency of reflexes [56], while the delay among components of the same signal is set such a way to minimize correlation among samples [57]. However, although the values of these parameters can be found in literature or can be easily estimated by computing the decorrelation time, their reliability is questionable. Indeed, while the latency of the interactions is usually estimated by opening closed loop circuits using surgical procedures and pharmacological challenges, thus being inadequate when closed loop reflexes are active, decorrelation time is an average parameter that cannot be optimal for any time scale, thus being inappropriate in presence of multiple temporal scales.

In addition, the proposed framework allows the comparison between two different techniques for the evaluation of complexity and causality inside the same class of MF approaches (i.e. LP and CE). The differences between LP and CE methods depend on the exploitation of two different functionals. While the LP approach exploited the correlation between observed and predicted data, the CE method quantified directly the uncertainty in the information domain. The comparison was carried out without altering the logic of coarse graining of the system evolution in the multivariate embedding space. Indeed, both LP and CE methods exploited the k-nearest-neighbor technique [27], [34]. The k vectors closest to the current one, regardless of their actual distance from it, formed the library of patterns utilized to build the conditional distribution of the future values (i.e. the distribution of the images of the k-nearest-neighbor vectors). The conditional distributions are utilized to set the predictor and assess the uncertainty as the variance of the prediction error in the case of the LP approach or as the average value of the Shannon entropy of the conditional distributions in the case of the CE method. Therefore, this framework provides a unique opportunity to compare different MF approaches under similar settings.

The framework proposes the contemporaneous evaluation of complexity and causality indexes. This simultaneous evaluation is important because the assessment of causality might be helpful to interpret variations of complexity with age in terms of physiological mechanisms. For example, since at REST the decrease of the strength of the causal link from RESP to HP combined with the invariable intensity of the causal relation from SAP to HP is incompatible with the decreased complexity of HP variability with age, we suggest that mechanisms other than cardiorespiratory coupling and cardiac baroreflex are responsible for the loss of complexity of the HP variability during senescence. During STAND the increase of the complexity of the HP variability with age was associated to a progressive decrease of the strength of the causal link from SAP to HP in presence of an invariable intensity of the causal relation from RESP to HP. Therefore, during STAND the observed increase of the complexity of the HP variability with age can be attributed to the progressive reduction of the effectiveness of the cardiac baroreflex. This finding suggests that cardiac baroreflex plays an important role in keeping low the complexity of HP variability and its impairment leads to an increase of the complexity of the cardiac control.

### Comparison Between MB and MF Approaches

Our findings pointed out differences between MB and MF approaches. These differences might be explained either in terms of the ability of the MF approach to account for potential nonlinearities present at the level of the dynamics of the series and/or at the level of the relations among different series, or in terms of different capabilities of the approaches in limiting the curse of dimensionality (i.e. the gradual decrease of the reliability of complexity and causality indexes with the embedding dimension [58] unavoidable in presence of a limited amount of data).

At REST, at variance of the MB technique, the MF approaches were able to detect the decrease of complexity of the SAP variability with age. The decline of the SAP complexity with age might be associated to the well-known gradual increase of sympathetic activity during senescence [59], [60] leading to the gradual increase of synchronization of sparse vasomotor activities at peripheral vascular levels [61]. Given the inherent nonlinear nature of synchronization it is not surprising to find out that the age-related modifications of SAP dynamics cannot be detected by a linear approach such as the MB one.

Moreover, at REST, again at variance with the MB approach, the MF techniques were able to detect the dependence of the intensity of the causal relation from RESP to HP on age. The presence of a causal relation from RESP to HP is the result of a nonlinear coupling between respiratory activity and vagal outflow determining the respiratory-related HP variations [22], [23], [62]. Since nonlinear mechanisms provides the basis of the coupling between respiratory activity and vagal outflow [22]. it is not surprising to find out that a linear MB approach cannot detect the gradual reduction of the strength of the causal link from RESP to HP with age.

The MB approach was also unable to identify the progressive decrease of the strength of the causal link from SAP to HP during STAND detected by the LP approach. The causal relation from SAP to HP during STAND is due to the activation of cardiac baroreflex in response of the orthostatic challenge [63]. Since it was observed that the likelihood of finding nonlinear interactions between SAP and HP variabilities increased in old subjects [64], again it is not surprising that a nonlinear MF approach such as the LP one is more powerful than a linear MB method in detecting the decreased efficiency of the cardiac baroreflex with age.

Regardless of the experimental condition MF approaches were unable to identify the progressive decrease of the intensity of the causal relation from HP to SAP. The causal link from HP to SAP is the consequence of two opposite tendencies [18]: 1) a negative relation of SAP on HP due to the diastolic runoff leading to a decrease of diastolic arterial pressure while lengthening HP and, thus, to a reduced SAP at the next cardiac beat in presence of an unaltered pulse pressure; 2) a positive relation of SAP on HP due to the Frank-Starling mechanism leading to an increase of SAP at the next cardiac beat due to the larger ventricular filling induced by the HP lengthening. The ability of the MB approach in detecting the effect of age on the causal link from HP to SAP can be explained by hypothesizing that this relation needs high dimensional embedding spaces to be perfectly unfolded. Unfortunately, MF approaches exploited in this study might be inefficient in presence of high dimensional dynamics, thus giving the reason for their incapacity in detecting the relation revealed by the MB approach. Indeed, MF approaches escape to the curse of dimensionality by drastically limiting the embedding dimension at the cost of reducing their efficiency in presence of high dimensional dynamics and imposing the enlargement of the data set to reliably explore higher dimensional spaces. Conversely, as a consequence of the less parsimonious strategy to construct the multivariate embedding space, the MB approach can be more efficient in presence of high dimensional dynamics.

### Comparison between LP and CE Approaches

Both MF approaches are parsimonious compared to the MB one, but the CE technique makes use of a smaller number of components than the LP one. Parsimoniousness is certainly helpful to limit the rate of false detection of causality when causality is not present (i.e. false positives), but it might increase the rate of false negatives (i.e. the probability of missing causality when a causal link does exist). This observation can explain the more frequent detection of age-related changes of complexity and causality indexes observed when the LP approach was applied compared to the CE one. It is worth noting that when both MF techniques detected a significant association of the parameters with age the sign of the relation is the same. The greater parsimoniousness of the CE approach might explain its inability to detect the progressive increase of complexity of the HP dynamics with age observed during STAND and to find out the progressive decrease of the strength of the causal link from SAP to HP and RESP to SAP with age during STAND. Both relations were identified by the LP method. However, since LP and CE techniques exploit different functionals it might happen that the differences between the two MF approaches might be related to the quantification of different aspects of dynamics as well.

## Discussion on the Experimental Findings

The experimental results about the age dependency of the complexity of the cardiovascular control can be summarized as follows: a) while HP complexity progressively decreases with age at REST, it gradually increases with age during STAND, thus suggesting an age-related impairment of the cardiac control and of the response of the cardiac regulation to stressors; b) SAP complexity gradually decreases with age at REST, thus indicating the progressive increase of sympathetic activity and/or modulation with age; c) no association between SAP complexity and age is observed during STAND, thus being indicative of a reduced responsiveness of the vasomotor control to stressors; d) RESP complexity is unrelated to age, thus demonstrating that respiratory control is preserved in elderly people.

The experimental results about the age dependency of the strength of the causal relations among cardiovascular variabilities can be summarized as follows: 1) while at REST aging preserves the strength of the causal link from SAP to HP along the cardiac baroreflex, during STAND a negative relation with age was found, thus indicating that in elderly individuals the cardiac baroreflex remains a fundamental reflex for homeostasis at REST but its efficiency in response to stressors is progressively lost with age; 2) at REST and during STAND the strength of the causal link from HP to SAP decreases with age due to the progressive exploitation of Frank-Starling mechanism at REST and the progressive increase of peripheral resistances during STAND; 3) regardless of the experimental condition the intensity of the causal relation from RESP to HP decreases with age, thus reflecting the increase of sympathetic tone and the uncoupling between respiratory activity and vagal outflow; 4) regardless of the experimental condition the intensity of the causal relation from RESP to SAP decreases with age as a probable result of the progressive increase of left ventricular thickness and vascular stiffness and of the gradual decrease of the respiratory-related HP variations; 5) the causal link from SAP to RESP is not affected by aging, as a likely result of the negligible role played by this pathway in short-term cardiovascular control; 6) the causal relation from HP to RESP is unmodified by aging due to the preserved nerve conduction velocity and neural processing time with age.

### Effect of Age on HP and SAP Traditional Parameters

We confirm that at REST the mean HP is unrelated to age [65], mean SAP progressively increases with age [65], and HP variance gradually decreases [4], [8], [66]. The tendency of SAP variance to increase with age at REST observed in [67] was found to be significant in this study. Several mechanisms have been advocated to explain these relations with age: i) the depressed pacemaker activity of sinoatrial node myocytes [68]; ii) the gradual augmentation of tonic sympathetic activity as measured from post-ganglionic sympathetic nerves directed to skeletal muscles [59], [60]; iii) the progressive increase of norepinephrine concentrations [36], [60], [69]; iv) the continuing decline of vagal modulation as assessed from the amplitude of respiratory sinus arrhythmia in the time or frequency domain [8], [36], [65], [70]; v) the gradual alteration of the adrenoceptor function [71]; vi) the progressive diminution of the responsiveness of the sinus node to sympathetic outflow [36], [65], [72]; vii) the steady decrease of BRS [36], [60], [65], [73]. The association between the BRS decline and the increase of the SAP variance with age observed at REST stresses the buffering role of baroreflex.

During STAND we confirm the positive dependence of HP mean and the negative relation of HP variance on age [36], [66], the positive correlation of SAP mean with age [73] and the lack of a linear relation between SAP variance and age [36], [73]. During STAND, at difference of REST, the BRS decline with age was not associated to a progressive increase of the SAP variance. This finding might be the consequence of the simultaneous decrease of the intensity of the causal relation from HP to SAP with age, thus leading to a situation of progressive HP-SAP uncoupling with age. These results have been explained by the reduced effect of the postural maneuver on the cardiovascular variables due to the diminished responsiveness of the sinus node to neural inputs in response to stressors [36], [65], [72], [74], to the reduced responsiveness of the vasculature to vasodilatator agents [75] and in reaction to stimuli [36], [65], [73], to the increase of peripheral resistances [65], and to the decreased cardiac baroreflex efficiency in response to the postural challenge [65].

### Effect of Age on Complexity Indexes

This study confirms the gradual decrease of the complexity of the HP dynamics with age detected at REST using a univariate approach [4], [6]–[9]. Since this finding was obtained by constructing a multivariate embedding space, we conclude that accounting for series more closely related to vascular and respiratory systems (i.e. SAP and RESP) was inessential for the quantification of complexity of the HP variability [11]. This finding appears to be robust because it was detected by all the proposed approaches. As a new finding STAND was associated with a progressive increase of the HP complexity with age. Since the HP complexity during STAND decreased [33], [76] as a result of the sympathetic activation and vagal withdrawal [73], [77]–[79], this finding indicates a reduced ability of the cardiovascular system to cope with the postural challenge with age. This finding confirms, at the level of the complexity of the cardiovascular control, the difficulty of old individuals to deal with sympathetic stressors such as exercise and/or orthostatic challenge [65], [80]. Therefore, we suggest the use of complexity analysis of HP series during orthostatic challenge to quantify the reduced ability of the cardiovascular control of elderly subjects to cope with stressors.

At REST the complexity of the SAP series was found to decrease with age. We speculate that the progressive increase of the sympathetic activity with age [59], [60] determines a decrease of the number of temporal scales observable in the SAP series in the low frequency band (i.e. from 0.04 to 0.15 Hz) by synchronizing mechanisms operating in this band (e.g. peripheral vasomotion) [61]. Conversely, the complexity of the SAP variability was unrelated to age during STAND. Since sympathetic activity progressively increased with age [59], [60] and STAND led to an additional sympathetic overactivation [77], [78], a progressive reduction of the SAP complexity could be expected during STAND. Since the expected progressive reduction was not found in this study, the lack of association between SAP complexity and age during STAND might be the result of the reduced responsiveness of vasculature to vasomotor sympathetic control with age [36], [65], [73].

Regardless of the experimental condition RESP complexity was unrelated to age. Therefore, it can be concluded that senescence does affect respiratory centers and brain stem level and, more generally, respiratory activity. Since respiration is a strong periodical function, even though with a certain degree of irregularity about the dominant frequency [33], it is not surprising to find out that this pattern is maintained during aging independently of the experimental condition (i.e. REST or STAND).

### Effect of Age on Causality Indexes

At REST we did not find any linear relation of the strength of the causal link from SAP to HP on age. This finding suggests that the intensity of the causal relation from SAP to HP was preserved in old individuals and, thus, in elderly people the relation from SAP to HP (i.e. the cardiac baroreflex) continues to play an important role in regulating cardiovascular variables. This result was confirmed by BEI. This result might appear surprising at the first sight because we found that BRS gradually fell with age [36], [60], [81]. Nevertheless, it is worth noting that the decrease of the gain of the relation from SAP to HP does not necessary imply a diminished strength of the causal link from SAP to HP because gain and strength measure different aspects of a relation between variables [15]. For example, during graded head-up tilt BRS progressively decreased [77], while the strength of the causal relation from SAP to HP progressively increased [23], [63], [82]. On the contrary, during STAND we observed a gradual reduction of the intensity of the causal relation from SAP to HP with age, thus suggesting a progressive reduction of the efficiency of the cardiac baroreflex control with age that was unveiled by a maneuver challenging cardiac baroreflex regulation (i.e. STAND). This finding was again confirmed by BEI, thus remarking the association between this traditional index [40] and state-of-art causality markers.

The causal relation from HP to SAP, i.e. the so-called mechanical feedforward pathway, forms with the cardiac baroreflex feedback the closed loop regulating HP-SAP interactions. This closed loop control was found active both at REST and during STAND [15], [63]. Since the mechanical feedforward pathway relies on the opposite actions of Frank-Starling mechanism and diastolic runoff, with a prevalence of the diastolic runoff in humans [18], the reduction of the strength of the causal link from HP to SAP with age could be observed in presence of the progressive balancing of the two opposite influences during senescence. At REST we can hypothesize that Frank-Starling mechanism gains importance with age in regulating HP-SAP variability interactions due to the diminished importance of the cardiac baroreflex [65], thus reducing the dominance of the diastolic runoff. During STAND the main mechanism underpinning the decrease of the strength of the relation from HP to SAP with age might be the increase of peripheral resistances progressively reducing the importance of the diastolic runoff during senescence [65]. The decreased strength of the causal link from HP to SAP combined with the diminished intensity of the causal relation in the opposite temporal direction (i.e. from SAP to HP) observed during STAND led to a situation of progressive HP-SAP uncoupling with age. This finding stresses further the gradual loss of the ability to deal with orthostatic challenge with age.

Another relevant finding of this study is the progressive decrease of the strength of the causal link from RESP to HP with age at REST. Since the relation from RESP to HP is the result of the continuous modifications of the membrane potentials of preganglionic vagal motoneurones and their responsiveness to stimulatory inputs imposed by respiratory activity [22], [62], the gradual decrease of the strength of the link from RESP to HP is likely to be the consequence of the gradual increase of tonic sympathetic activity with age [59], [60], [83], of the progressive vagal withdrawal leading to the progressive reduction of the respiratory sinus arrhythmia [8], [36], [65], [70] and of the gradual uncoupling between respiratory activity and the heart. This finding corroborates recent observations based on a transfer entropy approach using a fixed embedding dimension [49] and on a method quantifying phase synchronization between nonlinear models of the cardiac and respiratory oscillators [84]. This trend was not detected during STAND, thus suggesting that the limitation of the respiratory sinus arrhythmia induced by the sympathetic activation evoked by STAND did mask this phenomenon.

A significant negative linear association between age and the strength of the causal link from RESP to SAP was detected both at REST and during STAND. In a previous study we found a significant causal relation from RESP to SAP at REST and we demonstrated that it was maintained during orthostatic challenge [82]. The causal relation from RESP to SAP is the result of the mechanical influences on venous return and on large vessels due to respiratory-related modifications of intrathoracic pressure [85]–[87]. The observed decrease of the strength of the causal link from RESP to SAP might be a direct consequence of a less efficient modification of the stroke volume and a reduced effect of the intrathoracic pressure changes on large vessels resulting from the increase of cardiac and vascular stiffness with age [83], [88] due to the progressive left ventricular wall and large elastic artery intimal media thickening [83], [88]. Since the modification of the stroke volume at the respiratory rate depends also on the amplitude of the respiratory sinus arrhythmia, its well-known decline with age might contribute to the reduced respiratory-related variation of the diastolic filling and, thus, to the decline of the respiratory-related SAP changes with age.

None of the approaches was able to detect a linear association between age and the strength of the causal relation from HP or SAP to RESP both at REST and during STAND. Since previous studies suggested that a causal link from SAP to RESP was extremely unlikely in short-term cardiovascular regulation at REST and during postural change [15], [82], the uncorrelation between age and the strength of the causal relation from SAP to RESP was expected. Conversely, previous studies detected a significant causal link from HP to RESP as a result of the quickness of the neural cardiac influences at the respiratory rate compared to the slowness of the respiratory-related changes assessed according to respiratory inductive plethysmography exploiting thoracic belts [82], [89]. Since fast respiratory-related HP variations decreased with age, the lack of association between age and the strength of the causal link from HP to RESP might be an unexpected result. This surprising finding might be the consequence of the preservation of the nerve conduction velocity and neural processing time with age regardless of the experimental conditions.

### Limitations of the Study and Future Developments

The strength of causal relations was estimated according to the concept of Granger causality [12]. The Granger approach to the inference of causality has been chosen among others [90] due to its direct connection with the quantification of complexity based on predictability and information content. We advocate the practical exploitation of alternative definition of causality, including those implying an intervention (i.e. the physical manipulation of the cause to stimulate an effect) to elucidate on real data the dependence of the conclusions on the adopted paradigm and the difference between the assessment of causality based on spontaneous variability and physical interventions. Among possible extensions we encourage the use of techniques based on permutations of ordinal patterns [91], thus performing the analysis directly in the framework of symbolic dynamics. Since the null hypothesis of Normality of HP, SAP and RESP series was rejected in 41%, 54% and 88% at REST and in 46%, 34% and 82% during STAND a portion of the MB complexity might be the result of the relevant percentage of non-Normal series. The significant percentage of non-Normal distribution might have some impact on the MB causality indexes as well. Future efforts should be devoted to complete the automation of the MF analysis by proposing an automatic procedure for setting the few parameters, notably *k*, that still remain under control of the user. From a more physiological standpoint the description of the relation governing variability interactions should be rendered more complete by accounting for new, contemporaneously recorded, physiological quantities such as diastolic arterial pressure, left ventricular contractility, stroke volume and venous return. Moreover, instead of comparing young sedentary volunteers to elderly sedentary humans, future protocols should contrast the same control group with elderly athletes with preserved peak 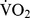 to clarify the role played by fitness deconditioning and separate it from that of senescence. Further applications should be devoted to make clear the role of diet in preserving complexity of cardiac regulation and the intensity of the causal relations among cardiovascular variabilities by comparing groups of people following different eating habits.

## Conclusions

This study proposed a compact framework for the assessment of complexity of cardiovascular variability series and causality of their interactions. The approach provided quantitative indexes that have been demonstrated helpful in elucidating the effect of age on cardiovascular control in humans. For example, results suggest that mechanisms other than cardiorespiratory coupling and baroreflex are responsible for the decrease of complexity of cardiac regulation with age at REST, while the impairment of the baroreflex is responsible for the increase of the complexity of the cardiac control with age during STAND. The proposed markers might be exploited in future clinical applications addressing the issue of monitoring the aging process and assessing the performance of countermeasures to the senescence of the cardiovascular control. The framework is particularly powerful because it allows the direct comparison between linear MB and nonlinear MF approaches and the minimization of the number of parameters needed to be set to perform complexity and causality analyses. Therefore, from a methodological standpoint, it is helpful to understand whether the exploitation of MF methods provides additional insights compared to a traditional simpler linear MB approach, and, from a more applicative standpoint, it favors user-independent applications and improves reproducibility of the results. The framework clearly demonstrates the importance of the MF methods especially in presence of nonlinear dynamics and nonlinearities in the interactions among series, the better reliability of the MB approach when the complexity of the interactions needs high dimensional embedding spaces to be fully unfolded, the greater statistical power of the LP approach compared to the CE one in detecting the age-related modifications of cardiovascular control, and the importance of computing causality indexes together with the more traditional complexity markers in the context of the evaluation of the senescence of cardiovascular regulatory mechanisms.
